# Iowa Dental Society

**Published:** 1872-10

**Authors:** 


### CORRESPONDENCE.

### Iowa Dental Society.

TABOR, IOWA.
Sept. 23, 1872.

DR. TAFT: *Dear Sir*—Yours of the 10th received. Our Society meets on the fourth Tuesday in July, annually. Next year it meets at Council Bluffs, Iowa. There are seventeen active members in our society. This year we met in the office of Drs. Charles and Pane, Omaha, Neb., July 23 and 24.

The first day was spent in an interchange of views on various subjects, explaining our modes of treating various cases, as inquiry was made by some one of the members.

The second day was spent in clinic. Dr. Pane filled a cavity in the first inferior right bicuspid, posterior approximal cavity. It was as well filled as though it had been done in New York or any of the big cities, and I think we have reason to be gratified that though we live in the far West we are not far behind in the skillful use of adhessive foil and the use of the "mallet."

I had the pleasure of taking my first lessons in the use of the mallet in the office of Prof. W. H. Atkinson and Dr. J. S. Lattimer, Ninth street, New York, and was the first to use it in the Missouri Valley. To-day there is not a first-class dentist in this State, so far as I know, who does not use them; and I claim to know the best dentists in *all parts of the State*.

Operative dentistry has made immense improvements in the West in the last six years, and it does not seem as though the next ten years could equal the improvements of the last

I wish mechanical dentistry could keep pace with it; but the people demand cheap material, and rubber has had a great run. The fusible bases are seldom used; they are too heavy. I have had to make over every one I have made, and use rubber or celluloid as a base instead. I am pleased with the latter, and use it almost to the exclusion of rubber. Gold and platinum are seldom or never used. Aluminum has seldom been used.

Respectfully yours,

